# How can neuroimaging facilitate the diagnosis and stratification of patients with psychosis?

**DOI:** 10.1016/j.euroneuro.2014.07.006

**Published:** 2015-05

**Authors:** Matthew J. Kempton, Philip McGuire

**Affiliations:** a Department of Psychosis Studies, Institute of Psychiatry, King׳s College London, UK; b Department of Neuroimaging, PO89, Institute of Psychiatry, King׳s College London, De Crespigny Park, London SE5 8AF, UK

**Keywords:** Early diagnosis, Psychosis, Biomarkers

## Abstract

Early diagnosis and treatment of patients with psychosis are associated with improved outcome in terms of future functioning, symptoms and treatment response. Identifying neuroimaging biomarkers for illness onset and treatment response would lead to immediate clinical benefits. In this review we discuss if neuroimaging may be utilised to diagnose patients with psychosis, predict those who will develop the illness in those at high risk, and stratify patients. State-of-the-art developments in the field are critically examined including multicentre studies, longitudinal designs, multimodal imaging and machine learning as well as some of the challenges in utilising future neuroimaging biomarkers in clinical trials. As many of these developments are already being applied in neuroimaging studies of Alzheimer׳s disease, we discuss what lessons have been learned from this field and how they may be applied to research in psychosis.

## 1 Introduction – the need for biomarkers

Despite significant advances in our understanding of the neurobiological basis of psychotic disorders, the assessment of patients with psychosis is still entirely based on a clinical interview, as it was when these disorders were first described over a century ago.

Patients with psychotic disorders such as schizophrenia usually experience prodromal symptoms 1 to 5 years prior to the first episode of frank psychotic illness (Hafner*,* 2000). This is described as an ‘at risk mental state’ (ARMS), as these individuals have a very high risk of developing psychosis, but only about 30% will go on to develop the illness (Fusar*-*Poli et al., 2012). There is evidence that clinical intervention at this stage may prevent them from developing psychosis (McGorry et al., 2002). However the key problem in the clinical management of ARMS subjects is that it is not possible, on the basis of clinical assessment, to reliably predict which individuals will subsequently develop psychosis. As a result, potentially preventative treatments are given to the entire sample, rather than just the subgroup that is destined to become psychotic. This is clinically inefficient and raises ethical concerns, as treatment may be given to subjects who would not have become psychotic in the absence of intervention. A biomarker that could identify the subgroup that will develop psychosis would enable preventative treatment to be targeted to those who needed it most.

The mainstay of treatment once a psychotic disorder has developed is antipsychotic medication. However, antipsychotic medication is ineffective in about a third of psychotic patients. The response in a given patient is unpredictable, and can only be determined through a lengthy process of trial and error, with the sequential evaluation of different drugs. A neuroimaging biomarker for treatment response might allow the unresponsive subgroup to be identified at presentation. They could then be offered clozapine, the only treatment that is effective in treatment resistant patients (Kane et al., 1988).

## 2 The search for imaging biomarkers

### 2.1 Multicentre studies

To identify robust neuroimaging biomarkers in psychosis for early diagnosis and patient stratification it is essential that neuroimaging studies are sufficiently powered. However, to date, sample size in neuroimaging studies of psychosis has been relatively small. For example in a recent meta-analysis of 283 structural MRI studies of patients with schizophrenia (Haijma et al., 2012), the mean sample size was 29 patients and 29 controls. However the largest effect size observed was *d*=0.5 to 0.6 (corresponding to the third ventricle, superior temporal gyrus, hippocampus, and fusiform gyrus volume). From the effect size one can calculate that to be sufficiently powered, a study would need to recruit at least 45 patients and 45 controls, which is more than 50% greater than the mean sample size. Thus the majority of structural neuroimaging studies of schizophrenia in the literature may not have been adequately powered. A lack of power is also an issue in imaging studies in other psychotic disorders such as bipolar disorder (Kempton et al., 2008), and is likely to apply to fMRI and DTI studies in psychosis, as these typically have smaller sample sizes than structural MRI studies (Minzenberg et al., 2009). It is important to clarify that the need for larger samples in neuroimaging studies does not preclude the possibility that information with clinical utility could be gleaned from a single subject (see machine learning section below). Schizophrenia is a heterogeneous illness that may encompass a number of different disorders each requiring a different approach to treatment. Acquiring large samples will be required to identify these subgroups. An analogous example is the successful stratification of different types of cancer, made possible by studying large groups of patients. The principle biomarkers for stratification are the morphology of cancer cells, genetic makeup of the cancer and particular proteins (Xing et al., 2005). In schizophrenia the successful stratification of the illness is likely to rely on biomarkers including genetics, neuroimaging and performance in neurocognitive tasks.

Increasing sample sizes in neuroimaging studies may be achieved by collecting data at multiple sites. This also ensures that identified biomarkers are determined from a representative patient population and are not specific to a local sample. This approach has been employed in the study of Alzheimer׳s disease by the Alzheimer׳s disease Neuroimaging Initiative (ADNI), which comprises 47 sites in USA and Canada (Jack et al., 2008). A challenge to multicentre MRI studies is between-centre heterogeneity in the acquisition of MRI data, which can lead to variations in estimated brain volumes. For example, we found a between-sequence variation of 4% in total grey matter volume (six T1 weighted MRI sequences described in (Kempton et al., 2011)). This amount is equal to the 4% reduction of total grey matter volume observed in the above meta-analysis of schizophrenia patients compared to healthy controls (Haijma et al., 2012). Thus if between centre/sequence heterogeneity is not minimised this can obscure reductions in brain volume observed in these patients. To address between centre heterogeneity the ADNI consortium developed a structural MRI sequence which produces an image with similar properties in terms of contrast, signal to noise ratio, and voxel size, independent of scanner model and manufacturer. The implementation of the ADNI MRI sequence has enabled the pooling of imaging data across sites, and the completion of an imaging study of Alzheimer׳s disease on a much larger scale (Schuff et al., 2009) than would have been possible at a single centre. In turn this has led to the identification and validation of structural neuroimaging biomarkers in Alzheimer׳s disease and their relationship to clinical outcome (Vemuri et al., 2009). There are additional challenges to multi-centre studies using functional MRI, as well as matching scanning parameters to obtain comparable sensitivity to brain activation, the presentation of stimuli (including considerations of language in international studies), recording participant׳s responses, and minimising head movement, all need to be standardised between centres.

In recent years, a number of prospective multicentre imaging studies have been initiated in psychosis research. The NAPLS study (North American Prodrome Longitudinal Study) (Cannon et al., 2008) and EU-GEI (European network of national schizophrenia networks studying gene-environment interaction) (van Os et al., 2008) both include multicentre neuroimaging of those at risk of developing psychosis. EU-GEI is collecting clinical, genetic and environmental data as well as ADNI structural MRI scans from individuals with an at risk mental state (ARMS). The ARMS study includes 11 centres in Europe, Australia and South America. As ARMS subjects are relatively difficult to recruit, and there are still only a limited number of specialist clinics globally which are able to recruit these individuals, multicentre studies provide an attractive way of significantly increasing sample size.

### 2.2 Longitudinal neuroimaging studies

Neuroimaging biomarkers identified in cross-sectional studies may be dependent on the stage of illness and modified by treatment; longitudinal studies are required to examine these effects in more detail. In this context, longitudinal designs are more powerful than cross-sectional studies, as each subject is effectively acts as their own control, removing variance due to baseline differences (typically large compared to changes seen over time). In addition, longitudinal studies of those at risk of psychosis may be able to identify novel biomarkers associated with the onset of psychotic illness as neuroimaging data may be acquired before and after psychosis occurs. Multiple scans over time also inform the continuing debate in the literature regarding the significance of progressive changes in brain structure (Kempton et al., 2010; Olabi et al., 2011) and whether these relate to the neuropathology of the disorder (Jarskog et al., 2005), antipsychotic treatment (Ho et al., 2011), or substance and alcohol abuse (Welch et al., 2011). There are particular challenges in conducting a longitudinal study, including sample attrition, upgrades of scanner hardware and software during the study, and even changes in the research staff running the project. Sample attrition rates depend on the length of follow-up, characteristics of the patient population and the research staff, and participants׳ opinions of the research project after their baseline assessment. Attrition rates are not always published; however rates up to 50% have been reported in longitudinal MRI studies of patients with psychosis (Thompson et al., 2009). Subject attrition leads to a reduction in statistical power due to reduced sample size. However a more serious problem is attrition bias, for example, if poorer functioning patients are less likely to attend follow-up, this will lead to an unrepresentative follow-up group. Therefore attrition should be minimised, and participants who do not attend follow-up should be compared to the included sample in terms of clinical and demographic characteristics. Neuroimaging scanners in research centres may have regular software and hardware upgrades which may have subtle effects on image contrast and geometric distortion. When processed with image analysis software this may lead to changes in regional brain volume, fMRI activation strength and differences in other modalities. Even changes in staff at the neuroimaging centre over time may lead to subtle effects, such as how the subject is positioned in the scanner. Although in a long term study scanner upgrades and staff changes may be unavoidable, it is important that there is no systematic difference between the patient and control group in regard to these changes. Longitudinal studies may provide better predictors of clinical outcomes in psychosis than a single scan. For example, (Cahn et al., 2006) found that the longitudinal change in MRI measures in the first year after the onset of psychosis predicted long term functional outcome, whereas the MRI data at baseline alone did not. However, the potential benefits of serial scanning have to be weighed against the increased logistical demands of longitudinal imaging studies, which may impact on the feasibility of the assessment in a clinical setting. Although longitudinal studies are known to be more powerful for detecting within subject change, it is unclear what difference this makes in terms of the required sample size for neuroimaging studies in psychosis. Therefore we investigated the example of detecting progressive changes (volume change greater than aging) in the volume of the lateral ventricles in patients with schizophrenia in a longitudinal versus cross-sectional design. In a longitudinal study this could be achieved by scanning patients and controls at baseline and at follow-up. In a cross-sectional study this could be determined by measuring the correlation between duration of illness and ventricle volume among patients. To calculate the required sample size to detect a significant effect, we conducted a numerical simulation where participants were assigned random demographic and clinical values from distributions matching typical imaging studies (mean age at baseline=29±6 years, duration of illness at baseline=7±4 years, follow-up period=3.5 years, (Kempton et al., 2010)). The effects of demographic and clinical variables on lateral ventricle volume were linearly modelled based on data published in previous studies (Haijma et al., 2012; Kempton et al., 2010; Kempton et al., 2011). From the simulation, we determined that a longitudinal study would require 64 patients and 64 controls to be sufficiently powered to detect progressive changes. In contrast a cross-sectional study would require 1260 patients for sufficient power to detect a correlation between ventricular volume and duration of illness. In this example a longitudinal design would be much more efficient because of large inter-subject variability in baseline ventricular volume and the relatively small effect of illness duration. The advantages and disadvantages of multicentre and longitudinal imaging studies are summarised in Table 1.

### 2.3 Predicting treatment response from neuroimaging data

Choosing the most suitable antipsychotic for a patient with first episode psychosis is often a process of trial and error. Ideally a clinician would be able to choose the best suited antipsychotic for a particular patient on the basis of investigations, without the need to evaluate a series of drugs over a lengthy period. A promising area of research is using neuroimaging assessments, prior to treatment to predict the response to antipsychotics. Kapur et al. (2000) demonstrated with PET (Positron Emission Tomography) that clinical response correlated with D2 receptor occupancy but that high occupancy >72% was associated with extrapyramidal side effects. Arango et al. (2003) reported that patients with higher regional brain volumes were more likely to benefit from clozapine treatment, and more at risk of side effects when treated with haloperidol. In a more recent study, Demjaha et al. (2012) found that patients who were responsive to antipsychotics had increased dopamine synthesis compared to both healthy controls and patients with treatment resistant schizophrenia. Using MR spectroscopy, Egerton et al. (2012) found that a poor response to antipsychotic medication in first episode patients was associated with elevated glutamate levels in the anterior cingulate cortex. Demjaha and Egerton had initially reported on separate samples but in a new publication (Demjaha et al., 2014) they assessed the same patient sample with both PET and MRS and reported that elevated glutamate levels within the anterior cingulate and normal presynaptic dopamine synthesis were both features of poor response to antipsychotic medication. The abnormalities identified by Demjaha et al. (2014) are biologically plausible as existing antipsychotics block the D2 receptor while the glutamate system is a proposed target for novel antipsychotics (Conn et al., 2009). Recent research has also used neuroimaging measures to predict the response to psychological therapies. Premkumar et al. (2009) reported that the response to cognitive behavioural therapy in patients with psychosis was associated with regional differences in grey matter volume, with an improvement in positive symptoms correlated with increased right cerebellar volume.

Ideally, when assessing whether neuroimaging measures can predict treatment response, neuroleptic naive patients should be recruited. This is particularly important, as there is increasing evidence that antipsychotic use may be associated with changes in grey matter volume over time (Ho et al., 2011), as well as with changes in regional brain function (Handley et al., 2013). However antipsychotic naive patients are difficult to recruit, and the majority of imaging studies in psychosis have included medicated patients. The OPTiMiSE study (Optimization of Treatment and Management of Schizophrenia in Europe, www.optimisetrial.eu) is a prospective multicentre clinical trial to optimise treatment in medication naive patients with schizophrenia. In this study, patients with first episode psychosis have an MRI scan at baseline and then start treatment with amisulpride. Patients with a poor response after 4 weeks are randomised to continuation on amisulpride or a switch to olanzapine. If patients continue to have psychotic symptoms they are offered clozapine. The OPTiMiSE trial uses the ADNI MRI protocol, ensuring that neuroimaging data can be pooled among the participating centres. The study will thus be able to examine the extent to which neuroimaging data can be used to predict antipsychotic response.

When assessing the contribution of neuroimaging, it is also informative to examine if other biomarkers or clinical variables have been successful in predicting treatment response. In relation to genetics, Zhang et al. (2010) reported in a meta-analysis that a polymorphism present in the gene that encodes the dopamine D2 receptor was associated with a significant change in clinical response to antipsychotics, while genome wide association studies (GWAS) which survey the entire genome have begun to report associations with antipsychotic response (McClay et al., 2011), see also review by Arranz and de Leon (2007). In terms of clinical variables, a meta-analysis by Perkins et al. (2005) demonstrated that a shorter duration of untreated psychosis was associated with an improved response to antipsychotics, while Crespo*-*Facorro et al. (2007) reported that an earlier age of onset predicted a poor response. Finally in a sample of 263 patients Green et al. (2004) found that patients with co-occurring substance abuse were less likely to respond when treated with either olanzapine or haloperidol. Thus neuroimaging may be one of a number of measures that can inform the prediction of response to specific antipsychotics.

### 2.4 Multimodal imaging

Multimodal imaging allows for one to investigate brain dysfunction using a range of measurement techniques within the same individual. Imaging in more than one modality (structural MRI, PET, fMRI, DTI, etc.) in the same patient may reveal detail about the neuropathophysiology of psychosis which could not be elucidated by one type of imaging alone. Multimodal imaging has also been successfully applied in Alzheimer׳s disease; structural MRI reveals prominent volumetric reductions in the hippocampus, PET scans with FDG (an analogue of glucose) show reduced metabolism, and PET scans with a ligand sensitive to beta-amyloids plagues (Pittsburgh compound B, PiB) reveal the distribution of this peptide in the brain (Jack et al., 2009). Multimodal imaging studies in patients with psychosis are most commonly MRI based techniques, as the subject can be scanned with a variety of sequences on the same scanner during a single session. However a limited number of centres which have access to both MRI and PET based scannings have been able to combine these data. Kalus et al. (2005) used 3 modalities namely structural MRI, diffusion tensor imaging (DTI) and magnetisation transfer imaging (MTI) to investigate the structure of the amygdala in patients with schizophrenia compared to healthy controls. Although no change in volume was found, diffusional anisotropy measured by DTI was reduced in patients and MTI parameters showed significant group differences. The latter two results may indicate abnormalities of intra-amygdaloid fibres and a reduction in white to grey matter ratio within the amygdala. Fusar*-*Poli et al. (2010) combined fMRI and PET datasets which measured brain activation in a working memory task, and dopamine synthesis, respectively. Data was acquired from 20 subjects with an at risk mental state compared to 14 healthy controls. The authors reported correlations between measured subcortical dopamine synthesis and activation in the prefrontal cortex among ARMS participants. In a recent study, Smieskova et al. (2012) combined structural MRI data with fMRI data in a group of patients with first episode psychosis, ARMS and controls. This study used a technique known as biological parametric mapping (Casanova et al., 2007), which allows voxelwise analysis of multimodal imaging data. Activation during a working memory task was reduced in a subgroup of ARMS subjects in the insular and prefrontal cortex which co-localised with a reduction in grey volume in the same regions. Sui et al. (2012) developed an alternative technique using canonical correlational analysis (CCA) and joint independent component analysis (ICA), to combine data from structural MRI, fMRI, and DTI scans. The methodology was applied to a large group of schizophrenia patients (*n*=97) and healthy controls (*n*=116) who had all been scanned using the 3 imaging modalities. Components discriminating patients and controls were found across the modalities, and a subgroup of components correlated with duration of illness. As the above selection of studies show there are a number of different combinations of imaging modalities and methodologies by which these data may be combined, making it challenging to summarise the findings from these studies. It is therefore encouraging that a method has been specifically developed to meta-analyse multimodal data. Radua et al. (2012) has developed a multimodal voxel-wise meta-analysis technique which has been applied to structural and functional MRI studies in patients with first episode psychosis. The technique allows the inclusion of independent fMRI and MRI studies meaning the meta-analysis is not restricted to studies which used a multimodal approach. The meta-analysis by Radua et al. identified both functional and structural abnormalities in the insula, superior temporal gyrus, medial frontal cortex and anterior cingulate. The meta-analyses also allowed the investigation of between study heterogeneity and by using meta-regression techniques determined that antipsychotic use was associated with structural abnormalities of the anterior cingulate and left insula.

Multimodal imaging may be an effective way of applying patient stratification prior to future clinical trials due to increased power to characterise subgroups from complementary measures. For example as reported above Demjaha et al. (2014) found that patients with elevated anterior cingulate glutamate levels (measured with MRS) and normal presynaptic dopamine synthesis (determined with PET) will have a poor response to antipsychotic medication. Thus prior to a clinical trial these patients could be identified and either excluded or treated with an adjunct medication. Using two other imaging modalities, namely structural MRI and electroencephalography (EEG), Molina et al. (2008) had shown that treatment resistance was associated with reduced frontal grey matter volume and a reduction in the intensity of the EEG P300 amplitude.

### 2.5 Machine learning approaches to classify individual patients

Research is often concerned with large groups of patients, while in the clinic decisions need to made based on information from a single patient. The requirement of large sample sizes referred to in the section above regarding multicentre studies may indicate that being able to apply this research to a single patient is not possible. However new approaches from the field of machine learning are increasingly being applied to neuroimaging data with an ability to classify a patient in terms of diagnosis based on their MRI data alone. The machine learning algorithm is presented with a training set of neuroimaging data in which the images are labelled by diagnosis, and after the training step the algorithm is able to classify entirely new neuroimaging data by diagnosis. The application of machine learning approaches in neuroimaging parallels an increase in the use of this tool, which is currently being applied to diverse datasets within medicine, such as the classification of survival rates in cancer from RNA expression (Olmos et al., 2012), identification of active tuberculosis from serum proteins (Agranoff et al., 2006) and the subdivisions of ADHD from neuropsychological data (Fair et al., 2012). A full technical description is beyond the scope of this paper; however the theory of machine learning and its application to neuroimaging is discussed in detail in two recent reviews (Kloppel et al., 2012; Orru et al., 2012). As machine learning approaches are a multivariate technique, the classification is not only based on regional differences in brain volume/activation, but also depends on covariance between brain regions. These techniques have recently been applied to neuroimaging in psychosis. Koutsouleris et al. (2009) used multivariate pattern classification of structural MRI data to successfully classify those with an at risk mental state and healthy controls. Furthermore based on the scans acquired at baseline they were able to use the technique to predict who would transition to psychosis compared to non-transitions and healthy controls with an accuracy of 88%. A method of pattern classification known as support vector machines (SVM) was applied to spectro-temporal data recorded using magnetoencephalography (MEG) by Ince et al. (2008). The authors reported 92% accuracy in classifying patients with schizophrenia compared to controls. The most discriminant signals used in the classification were located on the left side of the brain in the alpha, theta and delta frequency bands. Yoon et al. (2007) also used SVM but applied this to cortical thickness measures from structural MRI data. The accuracy of classification of patients with schizophrenia compared to healthy controls was at a similar level to the previous studies with an accuracy of 88–94% depending on the cortical lobe entered into the analysis. Costafreda et al. (2011) applied SVM classification to fMRI data of a verbal fluency task to distinguish patients with schizophrenia, bipolar disorder and healthy controls. Interesting although the SVM algorithm was successful at distinguishing patients with schizophrenia with an accuracy of 92%, the accuracy in identifying patients with bipolar disorder was lower with a number of these patients being misclassified as controls. In summary, machine learning approaches have shown promising results in determining psychiatric diagnosis from neuroimaging data. The highest accuracies in classification are around 90%, however this would need to substantially increase to be clinically useful. Conversely there has been some sceptiscm regarding the high levels of classification accuracy in neuroimaging studies, especially as recent DSM-5 field trials have reported a wide range of reliability for psychiatric diagnoses (Regier et al., 2013). The reliability for schizophrenia diagnosis was judged as ‘good’ with an associated kappa=0.46. As the training stage of machine learning depends on human diagnosis it may therefore be surprising that these algorithms are able to classify patents with an accuracy of 90%. However it should be noted that the DSM-5 field trials were designed to assess reliability in the real word clinic rather than a research institution, and clinicians did not use the Structured Clinical Interview for DSM (SCID) which is known to improve the reliability of diagnosis (e.g. kappa=0.65 for DSM-III-R schizophrenia) (Williams et al., 1992). Most of the imaging examples above compared patients with schizophrenia to healthy controls, however a more clinically useful test would be used to determine the diagnosis of a patient who was difficult to classify based on symptoms, for example determining if a patient with psychosis had bipolar disorder or schizophrenia. It is possible that adding other types of data to the machine learning algorithm such as neuropsychological, genetic and demographic information may lead to improved accuracy.

### 2.6 Translational neuroimaging: considerations of cost and regulatory approval of neuroimaging biomarkers

In terms of translating neuroimaging findings from the research arena to the clinic, it is important to consider the cost, image acquisition time, availability of imaging techniques in hospitals, and the skills needed to process and interpret data. For example PET imaging has been used with specific ligands to measure dopamine receptor availability in patients with schizophrenia (Howes et al., 2011) and amyloid plaques in patients with Alzheimer׳s disease (Jack et al., 2009). However these types of PET scans cost several thousand of Euros per patient, which may make it prohibitively expensive to use as a clinical tool. Diffusion tensor imaging has identified abnormalities in white matter tracts in patients with schizophrenia (Kanaan et al., 2009). However, at present the acquisition time and MRI hardware required mean that it is not easy for this technique to be adopted in some clinical centres. Structural MRI has an advantage as these types of images are routinely collected in hospitals. The post-processing of the images is complex but it is conceivable that an automated analysis pipeline can be developed and with the continuing rise in computing power, results would be available in a comparable time to having the image examined by a radiologist, but with the advantage that the results would be quantitative, standardised and not susceptible to inter-rater variability. If research demonstrated a robust neuroimaging biomarker for diagnosis or treatment response for patients with psychosis, a number of regulatory approvals would be needed before utilising the biomarker in clinical trials and in clinical diagnosis. No neuroimaging biomarker has reached this stage in psychosis research, but in Alzheimer׳s disease, automatic quantification of hippocampal volume is currently being considered for clinical trials (Jack et al., 2011). Approval by regulatory bodies such as the US Food and Drug Administration (FDA) and European Medicines Agency (EMA) would require comprehensive step-by-step validation and documentation of the technique (including the scanning protocol, data preprocessing and statistical analysis). Currently popular neuroimaging packages expressly advise against clinical applications in their software licence agreements (see http://surfer.nmr.mgh.harvard.edu). In terms of looking forward, developers of neuroimaging software should consider that their algorithms may need to be adapted in the future for clinical applications and to document and validate processing steps in preparation for this. As both clinical trials and fulfilling regulatory approval are costly, it is important to foster links with industrial partners who would be able invest in the development of neuroimaging biomarkers (e.g. IXICO; www.ixico.com).

#### 2.7 Translational neuroimaging in 2025

In this paper we have reviewed progress in the field of translational neuroimaging in psychosis; however how will the field look 10 years from now? We would like to see increased standardisation of MRI sequences allowing data from different centres and scanners to be easily combined which would be used in both multicentre and longitudinal studies. This would lead to the development of large public repositories of standardized MRI data of patients with psychosis paired with detailed demographic and clinical data. Access to these repositories would allow machine learning algorithms to be fine-tuned to predict treatment response and illness course in a subpopulation of patients. In parallel to the developments in neuroimaging, there is the hope that full genome sequencing in over half a million patients with schizophrenia will have identified hundreds of thousands of SNPs where each contribute to small effect of developing psychosis. Algorithms using polygenic scores could then be used to calculate psychosis risk for individuals as well as specifying which environmental factors should be avoided (such as cannabis use). For those identified as having a high risk of developing psychosis, regular MRI scans, proteomic analysis, clinical interviews and cognitive testing could be provided to monitor changes that might suggest the onset of a psychotic illness.

## 3 Conclusion

Neuroimaging research in psychosis has substantially advanced our understanding of the pathophysiology of the disorder. The challenge currently facing neuroimaging in this field is to translate these findings into mainstream clinical practice. The use of large scale multi-centre studies using standardised protocols, multi-modal imaging, the application of new analytical approaches like machine learning, and relating imaging measures to clinically meaningful outcomes will facilitate work towards this goal.

## Financial disclosure

None reported.

## Contributors

All authors contributed equally.

## Conflict of interest

None to declare.

## Figures and Tables

**Table 1:** Advantages and disadvantages of multicentre and longitudinal imaging studies.

	Multicentre studies	Longitudinal studies
Advantages	Increased sample size from pooling data from centres, allowing smaller effect sizes to be detected	More powerful design for detecting within subject changes
	Multicentre studies bring together expertise of data collection and analysis	Each subject acts as its own control which increases sensitivity
	Sample is more representative of the global patient population	Required to examine the effects of treatment and changes before and after psychosis onset
Disadvantages	Increased heterogeneity from variations in image acquisition, and patient population between centres	Participant attrition is a significant challenge particularly in imaging studies
	Variation in data quality from different sites	Upgrades in scanner software and hardware may change image contrast over the lifetime of the project
	Challenges in ensuring all investigators are sufficiently acknowledged in the work	Changes in research staff may lead to variations in the quality of data collection
